# Germline mutations in a clinic-based series of pregnancy associated breast cancer patients

**DOI:** 10.1186/s12885-021-08310-9

**Published:** 2021-05-19

**Authors:** Eleni Zografos, Anna-Maria Korakiti, Angeliki Andrikopoulou, Ioannis Rellias, Constantine Dimitrakakis, Spyridon Marinopoulos, Aris Giannos, Antonios Keramopoulos, Nikolaos Bredakis, Meletios-Athanasios Dimopoulos, Flora Zagouri

**Affiliations:** 1 Department of Clinical Therapeutics, Alexandra Hospital, School of Medicine, National and Kapodistrian University of Athens, 80 Vasilissis Sofias Avenue, 11528 Athens, Greece; 2 Department of Obstetrics and Gynecology, Alexandra Hospital, School of Medicine, National and Kapodistrian University of Athens, Athens, Greece; 3 Breast Center, Iaso Women’s Health Hospital, Athens, Greece

**Keywords:** Breast cancer, Pregnancy, Germline mutation, BRCA1, BRCA2, CHEK2, BRIP1

## Abstract

**Background:**

Pregnancy-associated breast cancer (PABC) defined as breast cancer diagnosed during gestation, lactation or within 1 year after delivery, represents a truly challenging situation with significantly increasing incidence rate. The genomic background of PABC has only recently been addressed while the underlying mechanisms of the disease still remain unknown. This analysis aims to further elucidate the frequency of PABC cases attributable to genetic predisposition and identify specific cancer susceptibility genes characterizing PABC.

**Methods:**

A comprehensive 94-cancer gene panel was implemented in a cohort of 20 PABC patients treated in our clinic and descriptive correlation was performed among the results and the patients’ clinicopathological data.

**Results:**

In the present study, 35% of PABC patients tested carried pathogenic mutations in two known cancer predisposition genes (*BRCA1* and *CHEK2*). In total, 30% of the patients carried *BRCA1* pathogenic variants. An additional 5% carried pathogenic variants in the *CHEK2* gene. Variants of unknown/uncertain significance (VUS) in breast cancer susceptibility genes *BRCA2, CHEK2* and *BRIP1* were also identified in three different PABC patients (15%). Not all patients carrying germline mutations reported known family history of cancer.

**Conclusions:**

Genetic testing should be considered as an option for PABC patients since the disease is highly associated with genetic susceptibility among other predisposing factors. Germline mutation identification may further modify PABC management approach and improve the prognostic outcome.

## Introduction

Breast cancer (BC) is the most frequent malignancy diagnosed in women; this observation generally applies to both the pregnant and the non-pregnant population [1, 2]. Pregnancy-associated breast cancer (PABC) is a complex situation that is commonly defined as breast cancer diagnosed during the period of pregnancy, lactation or within 12 months following delivery [3]. Up until today, PABC has been regarded as a rare entity since it accounts for only 0.2–3.8% of all breast cancer cases [4]. However, it has been well established that BC incidence increases with age, until the seventh decade [5]. Consequently, due to the social phenomenon of delayed childbearing, the incidence of PABC is currently on the rise in developing countries [6]. As far as developed countries are concerned, the application of non-invasive prenatal testing (NIPT) in all pregnant women today aiming to identify chromosomal abnormalities in the fetus, has significantly increased the detection of asymptomatic PABC patients [7, 8]. Additionally, the proportion of premenopausal women diagnosed with BC has increased substantially, with one in forty women diagnosed being under the age of 35 [9]. Taking all the above into account, PABC is expected to gain more scientific interest in the following years, in order to ensure that this rare and heterogenic population is offered the best individualized management for both maternal and fetal well-being.

Regarding the heredity of the disease, hallmark studies on familial BC have resulted in the identification of clinically relevant cancer susceptibility genes [10, 11]. Notably, most pregnancies occur under the age of 40, which is the age group in which BC has been more commonly associated with a positive family history and a higher occurrence of germline mutations [12]. In a study utilizing multiple-gene panel testing, 23% of breast malignancies in young women were related to germline mutations in known cancer predisposition genes such as *BRCA1/2*, *CHEK2*, *ATM* and *PALB2* [13]. In this setting, the latest international guidelines strongly recommend that genetic testing for high and moderate risk cancer susceptibility genes should be offered to every woman under the age of 50 with BC, thus encompassing PABC patients [14, 15]. Additionally, tumors in PABC patients bear distinct biological characteristics that deem them more aggressive, including higher grade and proliferation rates, advanced T stage at diagnosis, nodal involvement, greater prevalence of the triple-negative subtype (TNBC), and hormone receptor negativity [16]. Consequently, the genetic risk evaluation in this cohort of patients can have a substantial impact on PABC management and follow-up, offering a significant benefit not only to the affected mother to be, but also to family members [17].

Still, the genetic background of PABC remains an understudied field, despite the fact than in recent years the incorporation of multi-gene panel testing into clinical practice has enabled researchers to more accurately and cost-effectively estimate the proportion of cancers attributable to genetic predisposition and hereditary cancer syndromes [18]. Notably, numerous genes with non-silent mutations have been found to be differentially expressed between PABC and non-PABCpatient-derived tissues, as it was demonstrated in a recent systematic review published by members of our research group [19], implying a differential genomic background between pregnancy and non-pregnancy associated BC. In the current study, we applied multiple-gene panel testing to 94 cancer susceptibility genes aiming to identify the proportion of PABC cases attributable to genetic predisposition and to assess the prevalence of germline (i.e. inherited) mutations in PABC patients treated in our clinic.

## Materials and methods

In this cohort study, 20 women diagnosed with breast cancer during pregnancy or in the first year after delivery were enrolled. All participants were required to have completed the 18th year of age and to have attended the Breast Unit of the Obstetrical/Gynecological Clinic or the Department of Clinical Therapeutics of the National and Kapodistrian University of Athens, at the Alexandra Hospital in Athens, Greece. The sample was pooled from the hospital’s patient database according to the abovementioned inclusion criteria. The study was approved by the Institutional Review Board (IRB) of the participating hospital.

Participation was voluntary and once informed consent was granted by each participant, medical files of the patients were reviewed and researchers interviewed enrollees in person to collect demographic and clinical data, including age at diagnosis, family cancer history, prior genetic testing results, histopathologic evaluation (tumor stage, size, grade, lymph node status, hormone receptor, and HER2 status). Breast cancer diagnosis was established based on a combination of standard clinical, radiological and histological criteria [20]. Finally, a blood sample was collected from each PABC patient.

Subsequently, genomic DNA was isolated from whole blood using the QIAsymphony DSP DNA Mini Kit (Qiagen, Germantown, USA) and used to prepare indexed libraries to target the sequence of 94 cancer predisposing genes using the Trusight Cancer Panel – Νextera DNA Flex Pre-Enrichment Library Prep (Illumina, San Diego, USA). Libraries were qualitatively and quantitatively evaluated using a Fragment Analyzer (Advanced Analytical Technologies, Heidelberg, Germany) and sequenced on a MiSeq genetic analyzer (Illumina, Inc., San Diego, CA), according to the manufacturer’s protocols. Annotation was performed against the human reference genome GRCh38 using VariantStudio V.3 (Illumina, Inc., San Diego, CA). Based on the data generated from this software, alterations were identified as pathogenic when classified as disease causing or as variants of unknown significance (VUS) when evidence regarding their pathogenicity was either conflicting or limited [21]. The minimum base and amplicon coverage were 50×, and 100×, respectively, while the mean read depth was 182×. All sequenced variants were interpreted according to the recommendations of the American College of Medical Genetics and Genomics and the Association for Molecular Pathology [22].

## Results

We have screened 20 PABC patients, unselected for age or family history, for the presence of germline mutations. The detection rate of pathogenic mutations among the cases tested was 35% (7/20). Of those, six patients of our cohort had a pathogenic *BRCA1* mutation and one patient a *CHEK2* mutation. Of note, three PABC patients carried variants of unknown/uncertain significance (VUS) in three breast cancer susceptibility genes.

### Demographic characteristics

Detailed data concerning the major demographic variables and the pregnancy characteristics of the PABC subjects enrolled in this study are presented in Table 1, including age, ethnicity, BMI, time of diagnosis, and number of pregnancy. The age of diagnosis ranged from 26 to 45 years, with a mean age of 34 years. The majority of participants was Greek (18/20 = 90%), while one was of Albanian (5%) and one of Romani (5%) ancestry. According to the body mass index (BMI) chart, 35% of participants was classified as overweight and 25% as obese.

**Table 1:** Demographic Variables of the 20 PABC Patients Enrolled in the Study

	*Frequency*	*Percent*
**Age**		
25–29	2	10%
30–34	6	30%
35–39	9	45%
40 and older	3	15%
**Ethnicity**		
Greek	18	90%
Non-Greek	2	10%
**BMI**		
≤ 24.9 (Normal Weight)	8	40%
25.0–29.9 (Overweight)	7	35%
≥ 30.0 (Obese)	5	25%
**Time of Diagnosis**		
1st trimester (weeks 1–12)	1	5%
2nd trimester (weeks 13–26)	0	0%
3rd trimester (weeks 27-end)	8	40%
Post-partum (up until 12 months)	6	30%
N/A	5	25%
**Number of pregnancy**		
1st	4	20%
2nd	6	30%
3rd	1	5%
4th	1	5%
N/A	8	40%

### Histopathological types

Histopathological features of pregnancy-associated breast tumors are presented in Table 2. The most frequent histological type among PABC patients enrolled in our study was invasive ductal carcinoma (IDC) (90%). Interestingly, among them there were two cases of IDCs with medullary features, which fall into the basal-like molecular subtype [23]: one case concerned a 30-year-old woman diagnosed with PABC at the 28th week of pregnancy and the other was a case of bilateral disease with asynchronous onset at ages 41 (11 weeks pregnant) and 43 (non-pregnant). Concerning the latter case, the histopathological findings differed between the two diagnoses, since the pregnancy associated tumor exhibited medullary-like features, while two years later the same patient was diagnosed with metaplastic breast cancer of the other breast. Of the remaining PABC patients, there was one case of invasive lobular carcinoma (ILC) and one case of metaplastic carcinoma of the breast diagnosed simultaneously with ductal carcinoma. The majority of tumors was of high grade (80%), and pathological measurements regarding tumor size revealed that 75% of them were above 2 cm (T2, T3). As far as receptor status is concerned, 45% of PABC cases were ER negative, 60% HER2 negative and 50% of them PR negative; of note, 30% of the participants’ tumors were classified as triple receptor-negative breast cancer (TNBC). Axillary node infiltration was identified in 40% of cases.

**Table 2:** Histopathological Status of PABC Tumors

	Parameters	Numbers (%)
Histological type	IDC	18 (90%)
	ILC	1 (5%)
	Metaplastic	1 (5%)
Tumor grade	Low (grade I)	0 (0%)
	Intermediate (grade II)	4 (20%)
	High (grade III)	16 (80%)
Tumor size (T)	T1 (≤2 cm)	5 (25%)
	T2 (> 2 cm but ≤5 cm)	9 (45%)
	T3 (> 5 cm)	6 (30%)
ER	Negative	9 (45%)
	Positive	11 (55%)
PR	Negative	10 (50%)
	Positive	10 (50%)
HER2	Negative	12 (60%)
	Positive	8 (40%)
Ki-67	Low (< 15%)	6 (30%)
	Moderate (16–30%)	3 (15%)
	High (> 30%)	11 (55%)
Axillary Lymph Nodes	N0 (0 positive nodes)	12 (60%)
	N1 (1–3 positive nodes)	2 (10%)
	N2 (4–9 positive nodes)	2 (10%)
	N3 (≥10 positive nodes)	4 (20%)
Molecular subtypes	Luminal A	3 (15%)
	Luminal B (HER2 negative)	4 (20%)
	Luminal B (HER2 positive)	5 (25%)
	HER2-enriched	3 (15%)
	Triple negative	5 (25%)

### Germline mutation analysis

For those with a positive mutation result from genetic testing, Table 3 shows the identified germline mutations in cancer predisposition genes and the exact location of each mutation in the affected gene, in comparison with the family history of hereditary breast and ovarian cancer syndromes as well as other common cancers. Specifically, participant #553 has a germline mutation (*BRCA1: c.5328delC*) along with a family history of vulvar cancer, #750 has a known pathogenic mutation (*BRCA1: c.5212G > A*) with a family history of breast and ovarian cancer, #2754 is a carrier of a pathogenic mutation (*BRCA1: c.5251C > T*) with a family history of breast cancer, prostate cancer and basal cell carcinoma (BCC) of the skin, and finally PABC patient #2740 carries a germline mutation (*BRCA1 g.169527_180579del11052*) and has a history of endometrial and lung cancer. Interestingly, PABC subjects #964 and #749 were identified to have the same pathogenic mutation (*BRCA1: c.3700_3704delGTAAA*), however the former has no reported family history, while the latter has a family history of breast and endometrial cancer. Furthermore, one of our patients carried a known pathogenic *CHEK2* c.1100delC mutation, despite having no family history of cancer.

**Table 3:** Germline mutations identified in PABC cases

ID	Mutation (cDNA)	Mutation (protein)	Chromosome-Exon	Clinical Significance	Family History
*#553*	*BRCA1 c.5328delC*	*p.Thr1777fs*	*chr17 exon 21*	*Pathogenic*	*2nd degree relative: Vulvar cancer*
*#964*	*BRCA1 c.3700_3704delGTAAA*	*p.Val1234Glnfs*	*chr17- exon 11*	*Pathogenic*	*No*
*#749*	*BRCA1 c.3700_3704delGTAAA*	*p.Val1234Glnfs*	*chr17- exon 11*	*Pathogenic*	*2nd degree relatives: Breast, Endometrial cancer*
*#750*	*BRCA1 c.5212G > A*	*p.Gly1738Arg*	*chr17- exon 20*	*Pathogenic*	*2nd degree relatives: Breast, Ovarian cancer*
*#2754*	*BRCA1 c.5251C > T*	*p.Arg1751X*	*chr17- exon 20*	*Pathogenic*	*2nd degree relatives: Breast, Prostate cancer, BCC*
*#2740*	*BRCA1 g.169527_180579del11052*	*p.Gly1803_Tyr1863del11052*	*chr17- exons 23,24*	*Pathogenic*	*2nd degree relatives: Endometrial, Lung cancer*
*#1927*	*CHEK2 c.1100delC*	*p.Thr367MetfsX1*	*chr22-exon10*	*Pathogenic*	*No*
*#3227*	*BRCA2 c.8386C > T*	*p.Pro2796Ser*	*chr13 exon 18*	*VUS*	*No*
*#1045*	*CHEK2 c.1175C > T*	*p.Ala392Val*	*chr22- exon 20*	*VUS*	*No*
*#2122*	*BRIP1 c.2285G > A*	*p.Arg762His*	*chr17- exon 1*	*VUS*	*1st degree relative: Prostate cancer* *2nd degree relative: Breast cancer*

Note: Participants are identified by patient registry number along with their mutation and family history of cancer including hereditary breast and ovarian cancer syndrome (HBOC) – cancers, Basal cell carcinoma (BCC) of the skin, Variants of Unknown Significance (VUS), c. = coding DNA sequence, g. = genomic sequence, p. = protein sequence

Concerning the other *CHEK2* variant (*c.1175C > T)* that was identified in a 36-year-old patient with Luminal B- HER-2 negative PABC, it is classified as a variant of unknown/uncertain significance (VUS). Furthermore, another 37yo patient (#3227) carried a VUS in *BRCA2* (c.8386C > T), while having Luminal A-HER-2 negative BC and no cancer family history. Lastly, patient #2122 is a carrier of a VUS in the *BRIP1* gene; this PABC patient was diagnosed five months post-partum with Luminal B-HER-2 negative breast cancer at 32 years of age and had a positive family history of cancer, including a first- and a second- degree relative with prostate and breast cancer respectively.

## Discussion

In this descriptive study we have established the prevalence of *BRCA1* germline mutations in a group of 20 pregnancy associated breast cancer patients, which were selected regardless of family history. All in all, we identified seven PABC patients carrying known pathogenic germline mutations (35%), six of them in the *BRCA1* gene and one in the *CHEK2* gene. Additionally, variants of unknown/uncertain significance (VUS) in breast cancer susceptibility genes *CHEK2*, *BRCA2* and *BRIP1* were determined to be present in three different PABC patients (15%). To our knowledge, this is the first recent study aiming to investigate the germline mutation frequency and spectra in a clinic-based series of PABC patients.

The notably high frequency of *BRCA1* mutations found in the present PABC group (30%) places pregnant breast cancer patients in a high-risk setting. Previous studies, however heterogenous they are, report high *BRCA1* mutation prevalence in PABC patients, ranging from 11.4–19.6% [24, 25]. In this setting, PABC patients with germline mutations in BC susceptibility genes should be placed under close clinical monitoring, in accordance to the evidence-based clinical practice guidelines that have been developed to ensure the appropriate management for carriers of a *BRCA* pathogenic variant [15, 26]. This suggestion is consistent with the findings of the only similar study to ours published more than two decades ago by Johansson et al. (1998), who investigated the influence of pregnancy on the risk of developing breast cancer in germline *BRCA1/2* mutation carriers [27]. According to their results, more women with *BRCA1* mutations developed PABC and in the same context they proposed close monitoring of women with *BRCA1* familial mutations during and after pregnancy, thus substantiating our observations.

Nowadays, reports state that *BRCA1/2* mutations account for only approximately 50% of the identifiable germline cancer predisposition variants in BC patients [28]. In parallel, overwhelming evidence suggest that germline pathogenic variants in genes of intermediate penetrance, such as *ATM* and *CHEK2*, confer an increased risk of BC, and their analysis is encompassed in gene panels alongside the *BRCA1/2* genes [15, 29]. Notably, the checkpoint kinase 2 (*CHEK2*) gene is a tumor suppressor gene involved in cell cycle checkpoint regulation, DNA damage repair activation and apoptosis [30]. Given its essential role, *CHEK2* inherited pathogenic variants have been implicated in BC predisposition [31]. Of these, the founder mutation *CHEK2* c.1100delC is one of the most frequently identified among Northern Europeans [32], while it is considered to be less common in the Mediterranean region, including BC patients of Greek decent (0.16%) [33]. This observation does not coincide with our results, since we identified one carrier of the pathogenic *CHEK2* c.1100delC and another carrier of a *CHEK2* VUS among our PABC patients, highlighting the importance of further investigations of other mutations in order to unravel their contribution to PABC susceptibility.

Notably, multigene panel testing (MGPT) allows the sequencing of multiple genes simultaneously and has offered a cost effective and efficient way to assess cancer genetics in a phenotypically directed clinical setting [28, 34]. Multigene panel tests in general depict more clearly the proportion of breast cancers attributable to genetic predisposition, since they allow detection of even moderate or low penetrance genetic variants [35]. However, there are several issues to take into consideration regarding their use. Specifically, as gene panel testing options for breast cancer risk assessment continue to grow in variety, the specific multigene test that will be used should be chosen carefully. From a clinical perspective, choosing a gene panel that analyses a wide array of moderate or low penetrance genes may lead to the identification of genetic variants which are not medically actionable and thus do not exhibit the attendant cancer prevention benefits [36]. Additionally, multigene tests increase the likelihood of detecting a variant of unknown/uncertain significance (VUS). These VUSs add complexity that may cause difficulty for clinicians in making management recommendations and advising patients. In our study, a PABC patient carried a VUS in *BRIP1* (*BRCA1* interacting protein C-terminal helicase 1) which is a gene that contributes to the DNA repair function of BRCA1; the impact of this variant on molecular function and subsequent roles in cancer risk is uncertain. This is not a rare finding, since according to data available in ClinVar, 933 variants in *BRIP1* clinically classified as a VUS have been reported to date [37]. However, it is a noteworthy observation, since classification of VUSs identified in PABC patients can contribute to earlier detection and screening of breast cancer and, as data from multigene panel testing accumulates, eventually improve treatment options in PABC.

Limitations of the study include that large genomic rearrangements (LGRs), such as copy number variants (CNVs) are usually missed by multigene panel testing; hence they were not reported in our study. Furthermore, it is important to point out that our analysis focused on a small number of patients due to the rarity of the disease, deeming our results merely indicative and in need of further confirmation in a larger cohort of patients, in order to draw safe conclusions on whether a pathogenic variant of a gene can be associated with PABC. Lastly, since this is a descriptive study aiming to assess the prevalence of germline mutations in PABC, the limitations naturally include the absence of a comparison group. The approach utilized does not allow for causal statements; however, our results imply a noteworthy correlation and support the recommendation of at least *BRCA1* testing for all PABC patients, regardless of family history and age of diagnosis.

## Conclusions

In conclusion, this study highlights the high frequency of PABC cases attributable to genetic predisposition, indicating that genetic testing at an appropriate time is of great importance for this sub-group, since it might ensure that PABC patients receive the most appropriate treatment in respect to their specific needs and their sensitive situation. Additionally, our results imply a potential use of multigene panel testing that is not limited to young women at high risk but extends to patients with no family history. Interestingly, some of the PABC patients included in this study had family history of breast, ovarian, prostate, colorectal or endometrial cancer, while others reported no family history of malignancies, even though they had a positive result of genetic testing for pathogenic variants in high-risk genes. Taken together, these observations indicate that family history of cancer remains an important variable during decision-making about genetic testing among PABC patients, but it should not be the only criteria for patient selection if we want to assure that carriers will not be missed. Lastly, the use of multi-gene panel testing for hereditary breast cancer risk, not limited to *BRCA1/2* pathogenic mutations, is essential to increase the likelihood of detecting an underlying germline genetic component and achieve better PABC patient outcomes. Unfortunately, as already mentioned the incidence of pregnancy-associated breast cancer is expected to increase considerably in the years to come while the introduction of non-invasive prenatal testing has led to higher cancer detection rates in pregnant women [38]; therefore, further research in a larger cohort of patients is deemed necessary.

## Data Availability

All data generated or analyzed during this study are included in this published article.
